# Network-assisted target identification for haploinsufficiency and homozygous profiling screens

**DOI:** 10.1371/journal.pcbi.1005553

**Published:** 2017-06-02

**Authors:** Sheng Wang, Jian Peng

**Affiliations:** Department of Computer Science, University of Illinois at Urbana-Champaign, Urbana, Illinois, United States of America; University of Helsinki, FINLAND

## Abstract

Chemical genomic screens have recently emerged as a systematic approach to drug discovery on a genome-wide scale. Drug target identification and elucidation of the mechanism of action (MoA) of hits from these noisy high-throughput screens remain difficult. Here, we present **GIT** (**G**enetic **I**nteraction Network-Assisted **T**arget Identification), a network analysis method for drug target identification in haploinsufficiency profiling (HIP) and homozygous profiling (HOP) screens. With the drug-induced phenotypic fitness defect of the deletion of a gene, GIT also incorporates the fitness defects of the gene’s neighbors in the genetic interaction network. On three genome-scale yeast chemical genomic screens, GIT substantially outperforms previous scoring methods on target identification on HIP and HOP assays, respectively. Finally, we showed that by combining HIP and HOP assays, GIT further boosts target identification and reveals potential drug’s mechanism of action.

## Author summary

Chemical genomic screens have been developed to systematically explore compound-gene interactions with the goal of identifying new drugs and drug targets. Haploinsufficiency profiling and homozygous profiling screens measure the drug-induced growth sensitivities of deletion strains that are grown in the presence of a compound. Traditionally, putative target genes are ranked according to their importance for growth in HIP-HOP screens, since most sensitive strains often carry deletions of genes that are related to the drug target(s). Here, we present **GIT** (**G**enetic **I**nteraction Network-Assisted **T**arget Identification), a network analysis method for drug target identification. GIT scores a gene by combining its fitness defect with the screen outcomes of the gene’s neighbors in the genetic interaction network. We demonstrated that GIT significantly improves target identification and elucidates molecular and functional mechanisms of drug action.

## Introduction

Chemical genomic screens have been extensively used to discover functional interactions between genes and small molecular compounds *in vivo* [1–8]. Due to its short generation time, inexpensive cultivation, and facile genetics, the budding yeast *S. cerevisiae* has been widely used as a platform for chemical genomic screens to decipher proteins and pathways targeted by small molecular compounds [9–11]. In comparison to other approaches [12–15], yeast chemical genomic screens provide comprehensive and systematic genome-wide measurements of a complete set of deletion strains. When the target protein(s)’s functions are conserved throughout evolution, results of chemical genomic screens in yeast can be readily transferred to other species, including human [16–18]. There are two types of yeast chemical genomics assays: haploinsufficiency profiling (HIP) and homozygous profiling (HOP). A HIP assay consists of a set of heterozygous deletion diploid strains that are grown in the presence of a compound. Decreasing gene dosage of a drug target from two copies to one copy will result in increased drug sensitivity, or drug-induced haploinsufficiency [19]. Under normal condition, one copy of gene is adequate for the normal growth for diploid yeast. Haploinsufficiency can happen when a drug is added into the strain. Consequently, HIP experiments are designed to identify the relationship between gene haploinsufficiency and compounds. In contrast, a HOP assay measures drug sensitivities of strains with complete deletion of non-essential genes in either haploid or diploid strains. Because of the complete deletion, HOP assays identify genes that act to buffer the drug target pathway. The **fitness defect score (FD-score)** is widely used to predict drug targets by comparing the perturbed growth rates to those of a set of control strains [9, 10].

Recently, a large-scale Synthetic Genetic Analysis (SGA) study [20] showed that target’s genetic interaction profiles are highly correlated with the outcomes of chemical genomic screens, suggesting the possibility of combining genetic interaction profiles with chemical genomic screens for drug target identification [21–23]. A genetic interaction is measured as the difference between the experimentally measured double-mutant phenotype and the expected double-mutant phenotype [24]. A negative genetic interaction occurs when two genes have similar functions that compensate each other’s absence to support cell viability [25–27]. In contrast, a positive genetic interaction occurs when a mutation in one gene rescues the fitness defect associated with a mutation in another gene [28–30].

Intuitively, a gene’s genetic interaction neighbors are also modulated if the gene is targeted and perturbed by a compound. Hence, we can use genetic interaction neighbors’ FD-scores to assist the inference of drug targets on chemical genomic screens. To the best of our knowledge, the only previous attempt to combine chemical genomic screens with genetic interaction profiles is by computing the Pearson correlation coefficient between their outcomes [23]. A higher and positive Pearson correlation coefficient indicates a potential compound-target interaction because genetic perturbation is inherently similar to chemical perturbation. However, this approach often works poorly because the Pearson correlation coefficient is sensitive to the noise in high-throughput chemical genomic screens and SGA profiles. Moreover, these existing methods ignore the inherent differences between HIP and HOP assays. Since HIP and HOP assays are complementary, combining HIP and HOP should further improve target identification and enhance our understanding of compounds’ mechanisms of action (MoA) in a comprehensive way.

In this work, we introduce **GIT**, a novel **G**enetic **I**nteraction Network-Assisted **T**arget Identification scoring method for HIP-HOP screens. Due to the inherent similarity between genetic perturbation and chemical perturbation, it is possible to use genetic interaction neighbors’ chemical genomic profiles to assist drug target inference. Therefore, we adopt a network biology perspective to detect drug targets by its neighbors. We first constructed a weighted, signed genetic interaction network from SGA profiles. For HIP assays, GIT supplements a gene’s FD-score by the FD-scores of its neighboring genes in the genetic interaction network. If the FD-scores of its positive genetic interaction neighbors are high while the FD-scores of its negative genetic interaction neighbors are low, the gene is more likely to be a target. For HOP assays, GIT incorporates the FD-scores of long-range two-hop neighbors to identify drug targets, since HOP is more likely to prioritize genes that buffer the drug target pathway rather than the direct targets. By combining HIP and HOP assays using GIT, we observed further improvement in target identification. Extensive experiments on three genome-wide chemical genomic screens demonstrated that GIT substantially improves target identification in comparison with existing scoring methods. We also identified many novel compound-target interactions that are currently not in any curated database but are supported by literature. In addition to target identification, we further demonstrated that GIT can be used to reveal the mechanisms of action of compounds and uncover co-functional gene complexes.

## Methods

### FD-score

The fitness defect score (FD-score) is the log-ratio of the growth defect of a deletion strain in response to a compound treatment, relative to its growth under control conditions. For gene deletion strain *i* and compound *c*, the corresponding FD-score is defined as

$$\begin{array}{c} {\text{FD}}_{ic}=\log\frac{r_{ic}}{\bar{r_{i}}}, \end{array}$$ (1)

where *r*_*ic*_ is the growth defect of deletion strain *i* in the presence of compound *c*, and $\bar{r_{i}}$ is the average growth defect of deletion strain *i* measured under multiple control conditions without any compound treatment. FD-score reflects the sensitivity of a gene deletion strain to a compound treatment. Specifically, a negative FD_*ic*_ score means the growth fitness of the strain *i* in the presence of the chemical *c* should be weaker than that of the control without treatment. Therefore, a low, negative FD-score indicates a putative interaction between the deleted gene and the compound.

The FD-score does not consider epistasis or interactions among genes. However, recent studies indicate that the phenotype of a particular strain can be caused by the deletion of a genetic modifier of a neighboring gene that is responsible for the phenotype [9, 31–34]. Therefore, it is necessary to consider a gene’s neighboring genes for target identification.

### Genetic interaction network

We first obtained genetic interaction profiles of 5.4 million gene-gene pairs in yeast from a recent genome-scale Synthetic Genetic Array (SGA) study [20]. We then constructed a signed, weighted genetic interaction network based on these profiles. The edge weight *g*_*ij*_ between gene *i* and gene *j* in the genetic interaction network is defined as

$$\begin{array}{c} g_{ij}=f_{ij}-f_{i}f_{j}, \end{array}$$ (2)

where *f*_*ij*_ is the double-mutant growth fitness, and *f*_*i*_ is the single-mutant growth fitness of gene *i*. A negative genetic interaction refers to a more severe growth fitness observed than expected, with an extreme case being synthetic lethality, whereas a positive genetic interaction refers to double mutants with a less severe growth fitness than expected [35, 36].

### Network-assisted target identification in HIP assays

To identify drug targets in HIP assays, we introduce the GIT^HIP^-score, which combines a gene’s FD-score and the FD-scores of its neighboring genes in the genetic interaction network. For gene *i* and compound *c*, we define the GIT^HIP^-score as

$$\begin{array}{c} {\text{GIT}}_{ic}^{\text{HIP}}={\text{FD}}_{ic}-\sum_{j}{\text{FD}}_{jc}\cdot g_{ij}. \end{array}$$ (3)

**Fig 1:**
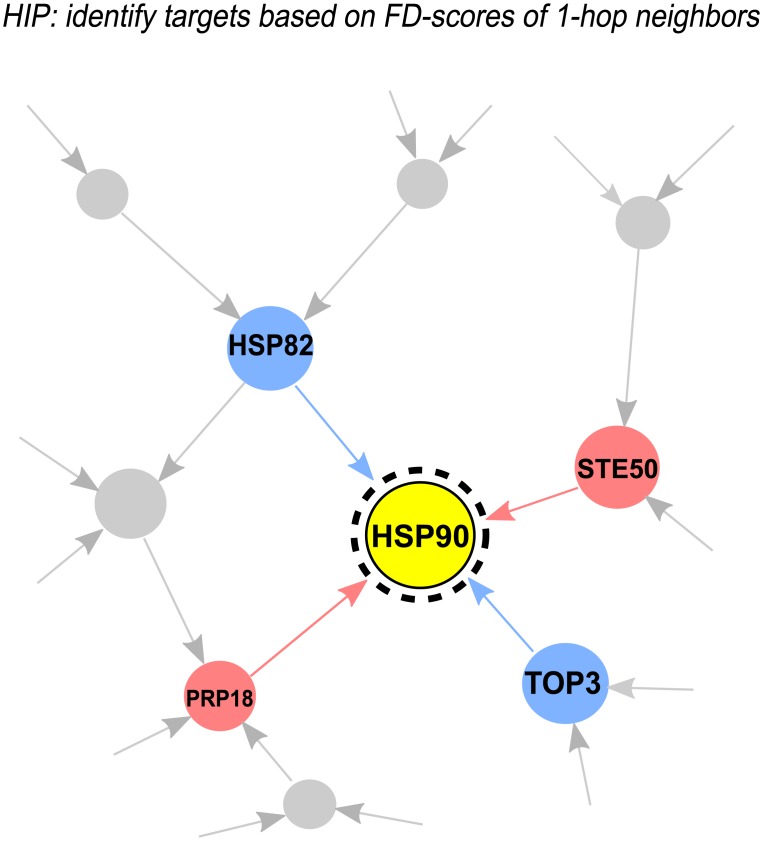
Illustration of how GIT identifies drug targets in HIP assays. Red (Blue) nodes indicate genes with high (low) FD-scores. Red (Blue) lines indicates positive (negative) genetic interactions. Yellow node indicates drug target. GIT supplements a gene’s FD-score by the FD-scores of its neighboring genes in the genetic interaction network. GIT identifies a gene as the target if the gene’s positive genetic interaction neighbors have high FD-scores and the gene’s negative genetic interaction neighbors have low FD-scores.

The GIT^HIP^-score considers two types of information of gene *i*: the FD-score of gene *i* and the FD-scores of gene *i*’s genetic interaction neighbors (Fig 1).

To account for different signs and strengths of genetic interactions, we compute a linear combination of the FD-scores of neighboring genes according to their genetic interaction edge weights to gene *i*. When gene *i* and gene *j* are a negative genetic interaction pair (*g*_*ij*_ < 0) and gene *i* is the target of compound *c*, it is very likely that we also observe a negative FD_*jc*_ value. This is because the deletion of one copy of gene *j* will make gene *i* more essential to the cell growth, according to the SGA assays. Likewise, if gene *i* and gene *j* are a positive genetic interaction pair (*g*_*ij*_ > 0) and gene *i* is the target of compound *c*, it is very likely that we observe a positive FD_*jc*_ value. This is because the deletion of one copy of gene *j* will make gene *i* less essential to the cell growth. Therefore, we designed the GIT score to integrate the information from the genetic interaction neighbors to increase the signal-to-noise ratio, thus improving the sensitivity of the target identification. A low GIT^HIP^-score indicates a potential compound-target interaction.

For target that cannot be accurately identified by the FD-score because of the noise in chemical genomic screens or neighboring gene effect [31], the GIT^HIP^-score corrects the target’s FD-score according to the FD-scores of its genetic interaction neighbors. Previous study proposed to identify essential genes in human cancer cell lines according to the expression profile of genetic interaction neighbors [37]. Specifically, one gene becomes more essential if its negative genetic interaction neighbors are inactive and its positive genetic interaction neighbors are active [38]. In this paper, we studied more direct phenotypic growth fitness from chemical genomics assays, instead of gene expression data. Accordingly, the GIT^HIP^-score can also be viewed as a conditional essentiality score since it captures the growth fitness of genetic interaction neighbors.

### Network-assisted target identification in HOP assays

Compared to HIP, HOP assays delete both copies of non-essential genes in either haploid or diploid strains. Therefore, the FD-score in HOP assays prioritizes genes that buffer the drug target pathway. In contrast to existing studies [9–11, 23], which apply the same scoring methods for HIP and HOP assays, we introduce a different network-assisted approach for HOP assays to tackle the inherent difference between HIP and HOP assays. Since genes with low FD-scores in the HOP assay are often close to or located in the drug target pathway, we extend our framework to consider the FD-scores of long-range two-hop genetic interaction neighbors.

To utilize long-range two-hop genetic interaction neighbors’ FD-scores, we first calculate the first-order GIT-score of each gene. We define the first-order GIT-score ${\text{GIT}}_{ic}^{1\text{st}}$ of gene *i* in the presence of compound *c* as

$$\begin{array}{c} {\text{GIT}}_{ic}^{1\text{st}}={\text{FD}}_{ic}-\sum_{j}{\text{FD}}_{jc}\cdot g_{ij}. \end{array}$$ (4)

We then use the first-order GIT-score to calculate the GIT^HOP^-score for target identification in HOP assays.

$$\begin{array}{c} {\text{GIT}}_{ic}^{\text{HOP}}={\text{FD}}_{ic}-\sum_{j}{\text{GIT}}_{jc}^{1\text{st}}\cdot g_{ij}. \end{array}$$ (5)

**Fig 2:**
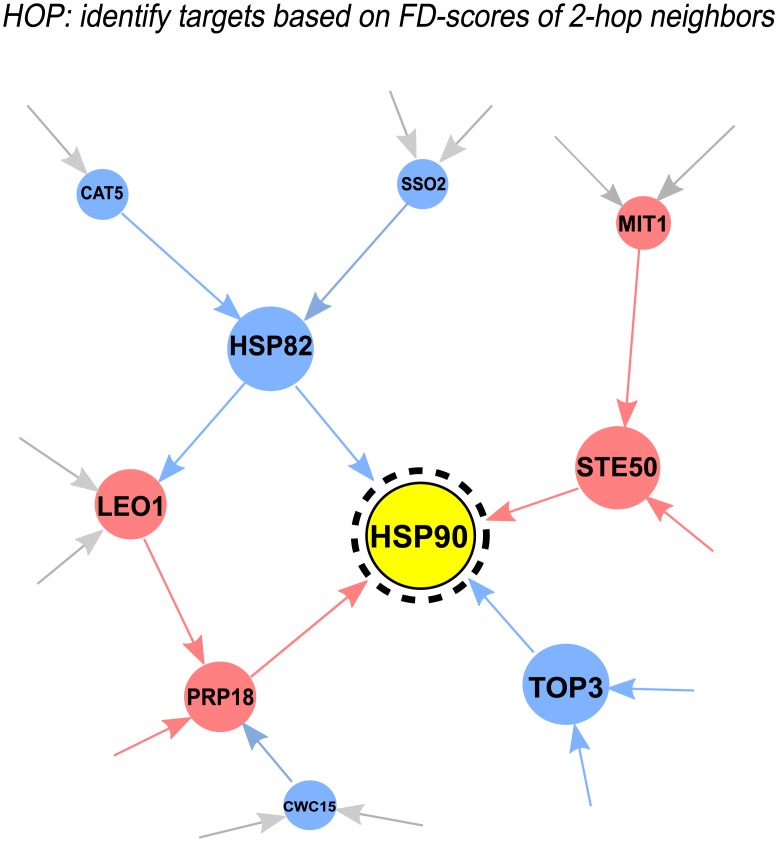
Illustration of how GIT identifies drug targets in HOP assays. Red (Blue) nodes indicate genes with high (low) FD-scores. Red (Blue) lines indicate positive (negative) genetic interactions. Yellow node indicates drug target. GIT supplements a gene’s FD-score by the FD-scores of its long-range two-hop neighbors in the genetic interaction network. These long-range two-hop neighbors capture the drug target pathway buffer effect in HOP assays.

The GIT^HOP^-score considers two types of information for each gene: the FD-score of the gene itself and the first-order GIT-scores of the gene’s genetic interaction neighbors (Fig 2). A low GIT^HOP^-score indicates a potential compound-target interaction.

While genes that act to buffer the drug target pathway may not be the one-hop neighbors of the target, the GIT^HOP^-score takes into account indirect neighboring genes in the genetic interaction network. Specifically, if the FD-scores of positively (negatively) weighted two-hop neighbors are high (low), the corresponding one-hop neighbors will have a significantly low GIT^1st^-scores. On the other hand, if the FD-scores of positively (negatively) weighted two-hop neighbors are low (high), the corresponding one-hop neighbors will have a significantly high GIT^1st^-scores. By correcting a gene’s FD-score according to the GIT^1st^-scores of the gene’s genetic interaction neighbors, the GIT^HOP^-score explicitly considers the FD-scores of two-hop neighbors, which capture the drug target pathway buffer effect in the HOP assay.

GIT^HOP^-scores can be viewed as the second-order GIT-score. It can be calculated by iteratively multiplying the FD-score vector with the genetic interaction network matrix. Although this scoring framework can be naturally extended to *k*th-order, we did not observe substantial improvement for *k* > 2 in our experiments. We showed the performance of using different *k* in S8 Fig.

### *ρ*-score

In addition to the FD-score, we compared our GIT-score with the *ρ*-score, which combines genetic interaction profiles with chemical genomic screens by computing the Pearson correlation coefficient between their outcomes [23]. *ρ*-score is proposed to leverage the inherent similarity between genetic perturbation and chemical perturbation. Both of them profiles the relative growth fitness when a gene *i* is knockdown by mutation or chemical compound. Thus if the genetic interaction profile of a gene *i* is positively correlated to the FD-score profile of a compound *c*, it means that the effect of mutating this gene *i* is similar to adding a compound *c*.

For gene *i* and compound *c*, the *ρ*-score is defined as

$$\begin{array}{c} \rho_{ic}=\frac{\sum_{k\in \text{ngh}(i)}\left({\text{FD}}_{kc}-\bar{{\text{FD}}_{c}}\right)\left(g_{ik}-\bar{g_{i}}\right)}{\sqrt{\sum_{k\in \text{ngh}(i)}{\left({\text{FD}}_{kc}-\bar{{\text{FD}}_{c}}\right)}^{2}\sum_{k\in \text{ngh}(i)}{\left(g_{ik}-\bar{g_{i}}\right)}^{2}}}, \end{array}$$ (6)

where ngh(*i*) denotes the genetic interaction neighbors of gene *i*. When calculating *ρ*_*ic*_, we excluded pairs of *k* and *c* if FD_*kc*_ is missing a value. A high, positive *ρ*-score indicates a potential compound-target interaction.

### Datasets

#### STITCH compound-target interactions and genetic interaction profiles

We obtained known compound-target interactions from the STITCH 4 database [39] as a benchmark to evaluate the performance of different target identification scoring methods. These compound-target interactions are built from heterogeneous data sources, including experiments, expert-curated databases, and literature mining. Each interaction has a combined score (between 0 and 1) representing the confidence. We excluded low-confidence interactions (combined score < 0.4), as suggested by the STITCH 4 database. We further excluded interactions predicted solely from putative homologs from other species. After filtering compounds that are not in any of the collected chemical genomics screens, we obtained 1472 compound-target interactions. We obtained genetic interaction profiles of 5.4 million gene-gene pairs in yeast from a recent genome-scale Synthetic Genetic Array (SGA) study [20].

#### Genome-wide HIP-HOP screens

We obtained three yeast HIP-HOP chemical genomic screens from [9–11]. For evaluation purposes, we only included compounds that had at least one STITCH compound-target interaction. The first screen from Hoepfner et al. 2014 has 4,146 and 4,921 deletion strains grown under 71 and 73 compound treatment conditions for heterozygous and homozygous deletion collections, respectively. The second screen from Lee et al. 2014 has 1,095 and 4,810 deletion strains grown under 382 compound treatment conditions for heterozygous and homozygous deletion collections, respectively. The third screen from Hillenmeyer et al. 2008 has 5,307 and 4,810 deletion strains grown under 333 and 162 compound treatment conditions for heterozygous and homozygous deletion collections, respectively.

### Experimental settings

We evaluated the performance of each scoring method based on two criteria: 1) the number of compound-target interactions that can be identified in the top *k* genes and 2) the number of compounds, at least one target of which can be identified in the top *k* genes.

For each criteria, we plotted a curve which describes the number of identified compound-target interactions (drugs for second criteria) against the rank of gene for each scoring method. The y-axis shows the number of compound-target interactions (drugs for second criteria) identified in the top *k* genes, where *k* is shown on the x-axis. We first calculated the area under the curve (AUC) of each scoring method.

Then we calculated a normalized AUC (nAUC) for scoring method X as

$$\begin{array}{c} {\text{nAUC}}^{X}=\frac{{\text{AUC}}^{X}}{{\text{AUC}}^{\text{FD}-\text{score}}}, \end{array}$$ (7)

where the AUC^FD−score^ is the AUC of the FD-score and the AUC^X^ is the AUC of the scoring method X. If the nAUC is larger than 1, then the corresponding scoring method is better than the FD-score method.

We denoted the nAUC obtained from the first criteria as nAUC_t_ and the nAUC obtained from the second criteria as nAUC_d_. Since one drug may have multiple targets, nAUC_t_ is more suitable for evaluation. Therefore, nAUC_t_ is the primary metric used for evaluation in this paper.

When calculating the GIT^HIP^-score and the GIT^HOP^-score, we only considered the top *q* positive genetic interaction neighbors with highest weights and the top *q* negative genetic interaction neighbors with highest absolute weights for each gene in the network. We set *q* to 100 in all three screens. We showed in S7 Fig that the performance of our method is not sensitive to different values of *q*.

## Results

### GIT substantially improves target identification in HIP assays

**Fig 3:**
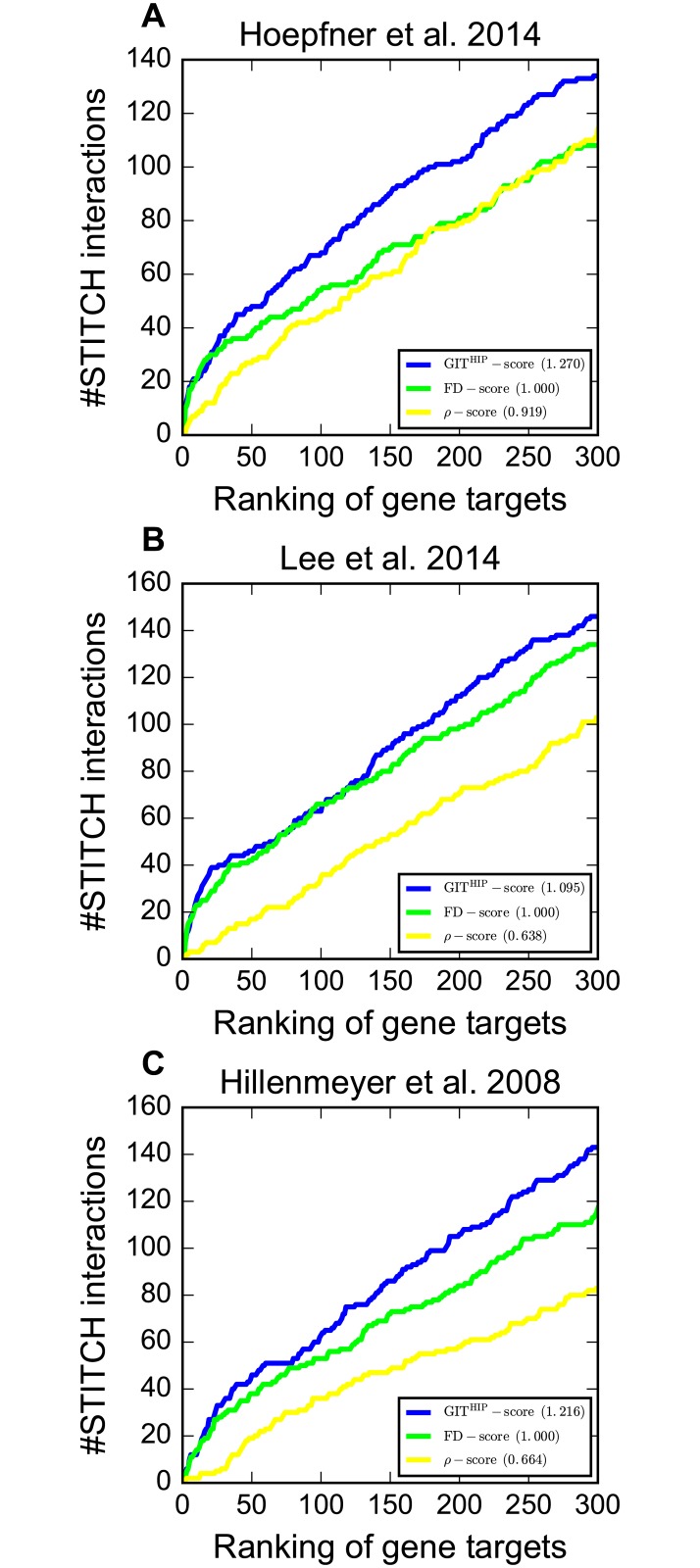
Comparison of GIT with other scoring methods in terms of nAUC_t_ in HIP assays on three chemical genomic screens. The y-axis shows the number of compound-target interactions identified in the top *k* genes, where *k* is shown on the x-axis.

To evaluate GIT in HIP assays, we performed large-scale target identification on three chemical genomic screens. The results are summarized in Fig 3 and S1 Fig. It is clear that our GIT^HIP^-score substantially outperforms other scoring methods on all three screens. For example, on Hoepfner et al. 2014 screen, GIT achieves 1.270 nAUC_t_, which is much higher than 1.000 nAUC_t_ of the FD-score. On Hillenmeyer et al. 2008 screen, GIT identifies 89 compound-target interactions in the top 150 genes, which is again substantially higher than the 75 compound-target interactions identified by the FD-score. Similar improvement was observed in terms of nAUC_d_ on all three screens.

To further understand how the GIT^HIP^-score achieves improved performance, we listed compound-target interactions that are ranked higher by the GIT^HIP^-score than by the FD-score in Table 1. For instance, the FD-score failed to identify the interaction between HSP90 and geldanamycin because of the large FD-score (2.01) of HSP90 in the presence of geldanamycin. In contrast, our GIT^HIP^-score successfully identified HSP90 as the target of geldanamycin by considering relevant HSP90’s negative genetic interaction neighbors (e.g., SGT1 with FD-score -8.77) and its positive genetic interaction neighbors (e.g., NSE3 with FD-score 0.68).

**Table 1:** Top compound-target interactions identified by GIT in HIP assays. We listed examples of compound-target interactions that are ranked higher by the GIT^HIP^-score than by the FD-score. We showed compound-target interactions that are identified in the top 100 genes by the GIT^HIP^-score.

Compound	Target	Rank by the GIT^HIP^-score	Rank by the FD-score
5-fluorouracil	GLT1	78	140
5-fluorouracil	DFR1	31	760
Curcumin	STT1	20	94
Cycloheximide	GSP1	64	513
Fenpropimorph	ERG11	33	5,567
Fenpropimorph	ERG2	26	56
Geldanamycin	HSP90	69	5,731
Hydrochloric Acid	END7	14	17
Hydroxyurea	SGS1	13	406
Latrunculin A	END7	13	56
Latrunculin A	PSL7	85	125
Methotrexate	DFR1	1	4
Radicicol	TOP3	79	961
Rapamycin	STT1	96	853
Rapamycin	TOR2	2	3
Rapamycin	LST8	4	30
Triclosan	OAR1	62	224

We further compared the GIT^HIP^-score with the *ρ*-score, which combines SGA profiles with chemical genomic screens via the Pearson correlation coefficient. We found that GIT substantially outperforms the *ρ*-score on all three chemical genomic screens (Fig 3 and S1 Fig). For instance, GIT achieves 1.270 nAUC_t_ on Hoepfner et al. 2014 screen, which is much higher than 0.919 nAUC_t_ of the *ρ*-score. Same as the observation in the previous work [23], the *ρ*-score performs consistently worse than the FD-score, possibly due to the fact that the Pearson correlation coefficient is sensitive to the noise in high-throughput chemical genomic screens and SGA profiles.

Finally, we examined whether other molecular networks also enable good target identification performance. Since previous work [40] has used protein-protein interaction network to identify essential genes in CRISPR screens, we hope to test if protein interaction network can also achieve good performance when is used to identify drug targets. Therefore, we compared the performance between protein interaction network and genetic interaction network on chemical genomics screens. We first obtained a physical interaction network (PI) of yeast proteins from BioGRID V3.4 [41]. There are no sign but only weighted edges in the obtained physical interaction network. We then used Eq 3 to calculate the GIT^HIP^-score(PI) based on this physical interaction network, where *g*_*ij*_ is the weight between gene *i* and gene *j* in the physical interaction network. We found that the GIT^HIP^-score has better performance than the GIT^HIP^-score(PI) (S2 Fig). Since the physical interaction network is unsigned, the GIT^HIP^-score(PI) inevitably prioritizes hubs which have a large number of neighbors. In contrast, by considering both negatively weighted edges and positively weighted edges in the genetic interaction network, our GIT^HIP^-score is not biased towards hubs and thus substantially enhances target identification performance.

### GIT substantially improves target identification in HOP assays

**Fig 4:**
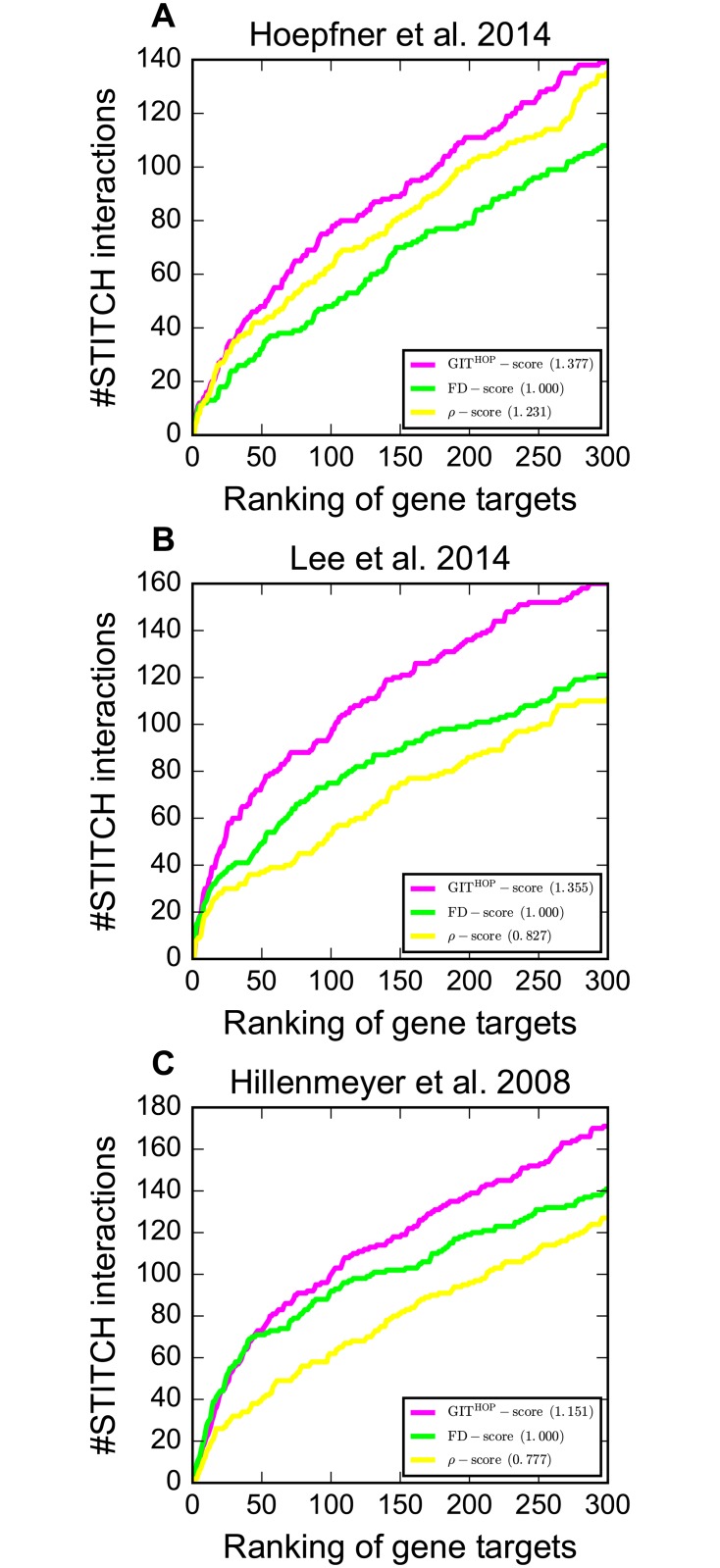
Comparison of GIT with other scoring methods in terms of nAUC_t_ in HOP assays on three chemical genomic screens. The y-axis shows the number of compound-target interactions identified in the top *k* genes, where *k* is shown on the x-axis.

We next performed large-scale target identification on three chemical genomic screens to evaluate GIT in HOP assays. The results are summarized in Fig 4 and S3 Fig. It is clear that our GIT^HOP^-score achieves the best target identification performance on all three chemical genomic screens. For instance, the GIT^HOP^-score achieves 1.355 nAUC_t_ on Lee et al. 2014 which is substantially higher than 1.000 nAUC_t_ of the FD-score. We listed compound-target interactions that are ranked higher by the GIT^HOP^-score than by the FD-score in Table 2. For instance, the FD-score fails to identify YSR2 as the target of sphingosine because of the close to zero FD-score (-0.017) of YSR2 in the presence of sphingosine. In contrast, the GIT^HOP^-score successfully identifies the interaction between YSR2 and sphingosine, mainly due to the high GIT^1st^-scores of YSR2’s positive genetic interaction neighbors (e.g., CSF1 with the GIT^1st^-score 1.60) and the low GIT^1st^-scores of YSR2’s negative genetic interaction neighbors (e.g., VBM2 with the GIT^1st^-score -3.00). The substantial improvement of GIT over the FD-score demonstrates the promising of utilizing two-hop genetic interaction neighbors’ FD-scores to identify targets in HOP assays.

**Table 2:** Top compound-target interactions identified by GIT in HOP assays. We listed examples of compound-target interactions that are ranked higher by the GIT^HOP^-score than by the FD-score. We showed compound-target interactions that are identified in the top 100 genes by the GIT^HOP^-score.

Compound	Target	Rank by the GIT^HOP^-score	Rank by the FD-score
Caffeine	TOR1	4	111
Camptothecin	RAD51	39	349
Fenpropimorph	ERG11	27	2,414
Fluconazole	ERG11	85	2,412
Hydrochloric Acid	GET3	79	278
Hydroxyurea	RAD51	20	1,751
Hydroxyurea	RAD52	82	1,396
Nocodazole	YOR29-09	62	393
Nocodazole	KAR9	26	256
Sphingosine	YSR2	2	1,981
Staurosporin	STT1	11	2,261

It is crucial to understand whether it is necessary to apply different scoring methods to the HIP assay and the HOP assay. To this end, we calculated the GIT^HIP^-score in the HOP assay based on Eq 3, where FD_*ic*_ (FD_*jc*_) is the FD-score of the HOP assay rather than the HIP assay. Consequently, the GIT^HIP^-score in the HOP assay corrects a gene’s FD-score only according to the FD-scores of its one-hop neighbors. Although the GIT^HIP^-score in HOP assays achieves better performance in comparison to the FD-score and the *ρ*-score, we noticed that it is consistently worse than the GIT^HOP^-score on all three screens (S4 Fig), suggesting the necessity of utilizing the FD-scores of two-hop neighbors to capture the drug target pathway buffer effect in the HOP assay.

We further investigated whether two-hop neighbors also enable better performance in HIP assays. We calculated the GIT^HOP^-score in HIP assays by using the FD-score of HIP assays in Eqs 4 and 5. Different from HOP assays, the GIT^HOP^-score does not obtain substantial improvement in comparison to the GIT^HIP^-score in HIP assays, reflecting the inherent differences between the HIP assay and the HOP assay.

Finally, we examined whether using the physical interaction network enables good target identification performance in HOP assays. We used Eqs 4 and 5 to calculate the GIT^HOP^-score(PI) based on the obtained physical interaction network, where *g*_*ij*_ is the edge weight between gene *i* and gene *j* in the physical interaction network. We compared the GIT^HOP^-score(PI) with the GIT^HOP^-score in HOP assays (S4 Fig). Similar to our observation in HIP assays, we found that using the genetic interaction network has an overall better performance than using the physical interaction network in HOP assays.

### Combining the HIP assay with the HOP assay further improves target identification

Since the HIP assay and the HOP assay are inherently different, we then studied whether these two assays can be combined for improving target identification. We noticed that either using the GIT^HIP^-score from the HIP assay or using the GIT^HOP^-score from the HOP assay identifies compound-target interactions that are not identified by the other (S5 Fig). For example, in the Hoepfner et al. 2014 screen, the GIT^HOP^-score identifies 11 compound-target interactions that are not discovered by the GIT^HIP^-score. Since the HIP assay and the HOP assay are complementary, we sought to combine them in order to further enhance the target identification performance.

**Fig 5:**
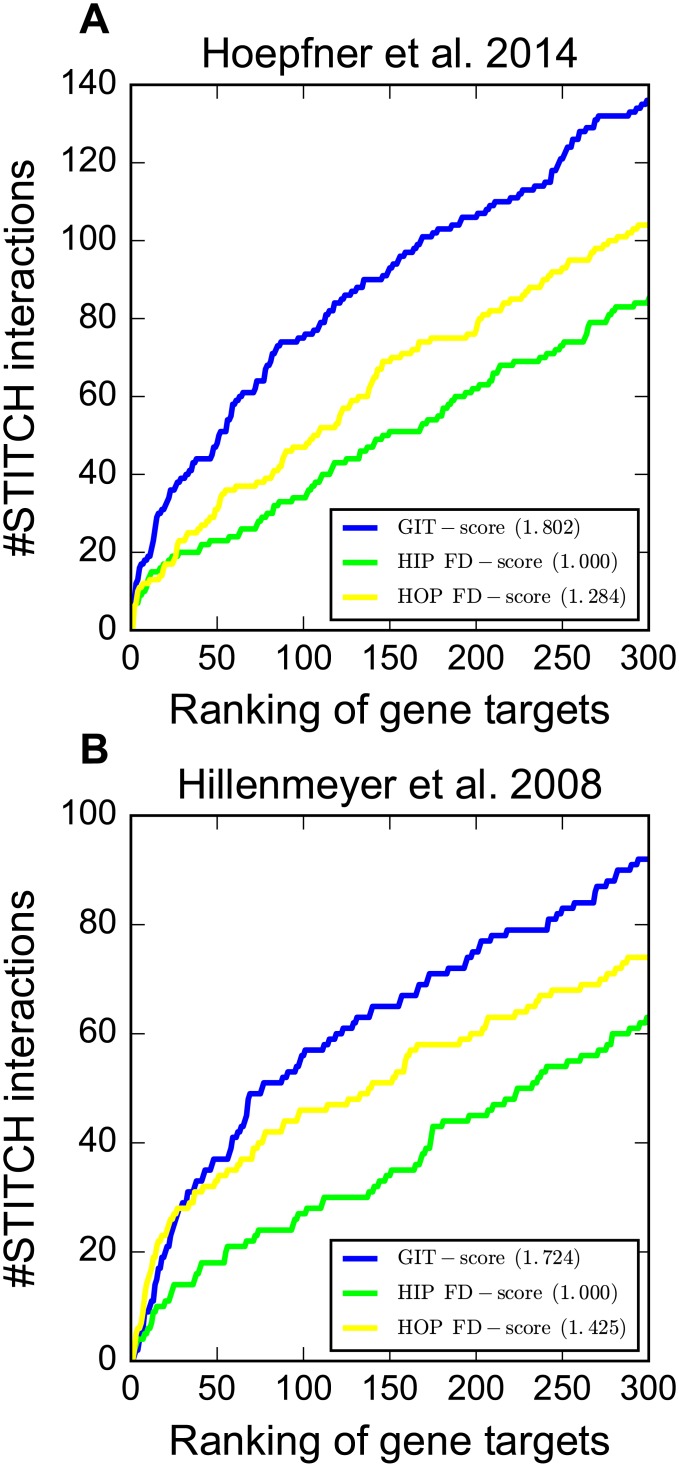
Comparison of GIT with other scoring methods in terms of nAUC_t_ on two chemical genomic screens. The y-axis shows the number of compound-target interactions identified in the top *k* genes, where *k* is shown on the x-axis. The GIT^HIP^-score is calculated by using the FD-score from the HIP assay. The GIT^HOP^-score is calculated by using the FD-score from the HOP assay. To calculate nAUC_t_, we divided the AUC of each method by the AUC of the GIT^HIP^-score.

We proposed a combined scoring method which takes the average of the *z*-score by the GIT^HIP^-score from the HIP assay and the *z*-score by the GIT^HOP^-score from the HOP assay. Averaging these two scores can be viewed as boosting two weaker scoring methods to create a more robust and better scoring method. We denote this score as the GIT-score. Since Lee et al. 2014 only measures the FD-scores of the heterozygous strains of essential genes and the homozygous strains of nonessential genes, there is no overlapping genes between the HIP assay and the HOP assay in this screen. Therefore, we evaluated the GIT-score on the other two chemical genomic screens. We observed that the combined GIT-score is substantially better than both the GIT^HIP^-score and the GIT^HOP^-score (Fig 5). For example, the GIT-score achieves 1.802 nAUC_t_, which is much higher than 1.284 nAUC_t_ of the GIT^HOP^-score and 1.000 nAUC_t_ of the GIT^HIP^-score on the Hoepfner et al. 2014 screen.

### Statistical assessment of using genetic interaction network in GIT

Since drug targets are likely to be enriched with high-degree nodes in the network [42], the improvement of GIT may be caused by its ability to prioritize these high-degree nodes instead of utilizing neighboring gene’s FD-scores. We then examined whether the improvement of GIT comes from prioritizing high degree nodes. We first constructed a large set of random networks according to the following procedure. For each node, we replaced each of its neighbors to another random node in the network while keeping the same edge weight and sign. Hence, each node in the new random networks has the same number of positive weighted neighbors and negative weighted neighbors as in the original genetic interaction network. We then used these networks to calculate the GIT-score, where *g*_*ij*_ is the edge weight between gene *i* and gene *j*. We calculated an empirical *p*-value according to the number of random networks that have better performance than the genetic interaction network when used to identify targets. Since each node in the new random networks have the same degree as it does in the original genetic interaction network, this empirical *p*-value tests whether the improvement of GIT is achieved by identifying high-degree nodes. We obtained significant empirical *p*-values on both screens (empirical *p*-value < 0.009 on Hoepfner et al. 2014; empirical *p*-value < 0.018 on Hillenmeyer et al. 2008). Therefore, we found that GIT on these random networks is significantly worse compared to GIT on the original GI network. This demonstrates that the improvement comes from correcting each gene’s FD-score with its neighbors’ FD-scores rather than the network topology only.

### GIT elucidates established mechanism of action of compound

**Fig 6:**
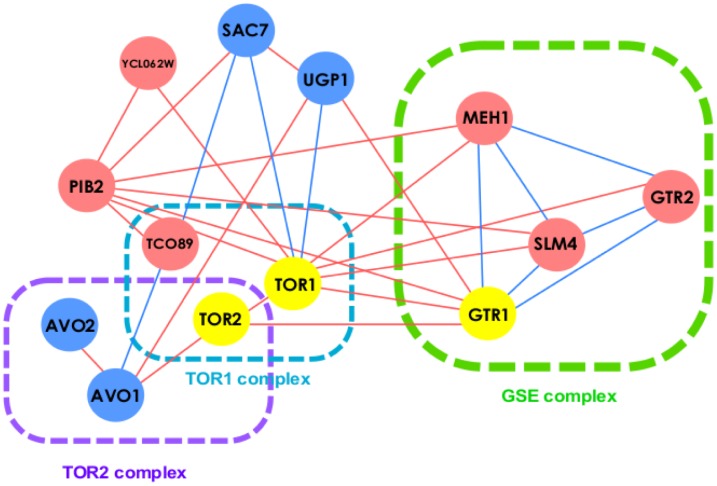
GIT elucidates MoA of caffeine. Green lines indicate positive genetic interactions. Red lines indicate negative genetic interactions. Red nodes (e.g., GTR1) are genes with low FD-scores in the HIP assay and the HOP assay. Green nodes (e.g., SAC7) are genes with high FD-scores in the HIP assay and the HOP assay. TOR1 and TOR2 are established targets of caffeine. The GIT-score successfully identifies their interactions with caffeine by using neighboring genes’ FD-scores. TOR1 complex, TOR2 complex, and GSE complex are identified as crucial cellular components that interact with caffeine.

To understand how GIT achieves the substantial improvement, we studied how GIT-score elucidates the compound’s MoA. We examined caffeine, which is a distinct, small molecular inhibitor of TOR complex [43, 44]. We analyzed GIT’s performance based on the Hoepfner et al. 2014 chemical genomic screen, which is the most recent screen among all three screens. We first noticed that the FD-scores of TOR1 in the HIP assay and the HOP assay are 0.079 and -1.827 in the presence of caffeine, respectively. Consequently, the FD-score fails to identify TOR1 as the very top target candidate. In contrast, our GIT-score successfully identifies TOR1 as the target of caffeine, mainly due to the high FD-scores of its positive genetic interaction neighbors SAC7 and UGP. The high FD-scores of SAC7 (1.75) and UGP (2.38) indicate that their positive genetic interaction neighbor TOR1 is inhibited by caffeine. Moreover, most of TOR1’s negative genetic interaction neighbors have substantially low FD-scores (e.g., GTR1 has -4.52 FD-score). We show the genetic interaction neighbors of TOR1 in Fig 6. We can see that TOR1’s negative genetic interaction neighbors have low FD-scores, whereas its positive genetic interaction neighbors have high FD-scores. Even though TOR1 does not have a substantially low FD-score, GIT still accurately identifies TOR1 as the target of caffeine by correcting TOR1’s FD-score according to its genetic interaction neighbors’ FD-scores.

Notably, the GIT-score also identifies TOR2 as a target of caffeine. Although the GIT-score ranks TOR2 lower than TOR1, it ranks TOR2 higher than the FD-score does. We noticed that it is difficult to identify TOR2 only according to one-hop genetic interaction neighbors’ FD-scores. Both TOR1 and AVO1 have close to zero FD-scores. GTR1 has a substantially low FD-score, but the genetic interaction edge weight between GTR1 and TOR2 is much lower than the one between GTR1 and TOR1. Nevertheless, the GIT-score still identifies TOR2 as a target of caffeine through the GIT^1st^-scores of TOR2’s genetic interaction neighbors (e.g, GTR1, TOR1 and AVO1).

Fig 6 not only elucidates how the GIT-score identifies the targets of caffeine, but also reveals the underlying MoA of caffeine. We can see that there are three major functional complexes that interact with caffeine. Both the TOR1 complex and the TOR2 complex are established cellular components that are affected by caffeine [43, 44]. GSE complex, along with PIB2 and YCL062W, are all associated with vacuolar membranes which play important roles in the TOR2 complex [45].

### GIT discovers novel compound-target interactions

We then investigated whether those novel compound-target interactions that are discovered by the GIT-score can be supported by existing literature. The output of GIT is a score for each compound-gene pairs. The top ranking genes are the potential targets of each drug. According to the average number of targets of each compound, we proposed to use an empirical p-value 0.001 as the cut-off values of significant compound-target interactions. Here, for each compound, we examined the top five genes that were predicted to be potential targets by the GIT-score. We listed the novel compound-target interactions that are supported by literature in Table 3. To show the advantage of using the genetic interaction network, we only listed targets that cannot be identified by the FD-score. For example, the GIT-score successfully identifies the interaction between 5-fluorouracil and SSF1. This interaction is verified by a haploid yeast knockout strains screen [46]. In contrast to the FD-score which fails to identify SSF1, the GIT-score identifies this interaction according to the high FD-scores of SSF1’s positive genetic interaction neighbors (YLR407W, TSL26) and the low FD-scores of SSF1’s negative genetic interaction neighbors (RRP6, YMR268W-A). The GIT-score also identifies POL32 as the target of camptothecin, which is verified by a recent cross-species chemical genomics profiling [47]. Again, the FD-score of POL32 in the presence of camptothecin is not significantly low (-0.38 in the HIP assay and -1.38 in the HOP assay). The GIT-score identifies POL32’s interaction with camptothecin through the high FD-scores of its positive genetic interaction neighbors (VPS39, BTS1 and RPL13A) and the low FD-scores of its negative genetic interaction neighbors (YCL060C, XRS1 and RTT110).

**Table 3:** Novel compound-target interactions identified by GIT. We listed compound-target interactions that are identified by the GIT-score but are currently not in any curated database. All these interactions are supported by literature. For each identified targets, we also listed its positive genetic interaction neighbors that have high FD-scores and its negative genetic interaction neighbors that have low FD-scores.

Compound	Target	PMID	Positive neighbors	Negative neighbors
5-fluorouracil	SSF1	18314501	YLR407W, TSL26	RRP6, YMR268W-A
Bafilomycin A1	DOR1	22470510	RPP1A, RTT103	SYS1, DLP2
Caffeine	SLT26	16729036, 16738548	HUR1, MRM2	SWM2, SFB2
Camptothecin	POL32	21179023	VPS39, BTS1, RPL13A	YCL060C, XRS1, RTT110
Curcumin	SEC37	21908599	SPB8, SCS1	DLP2, GET3

### GIT groups genes into co-functional gene complexes

**Fig 7:**
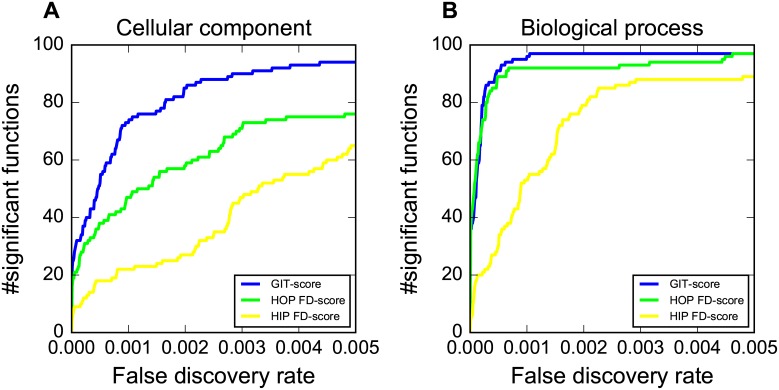
GIT identifies co-functional gene complexes. The y-axis shows the number of co-functional gene complexes that can be identified by the GIT-score, the FD-score from the HIP assay, and the FD-score from the HOP assay by using false discovery rate *r*, where *r* is shown in the x-axis. (A) shows the biological process category. (B) shows the cellular component category.

In addition to understanding of compound’s MoA, we studied whether the GIT-score can be used to identify co-functional gene complexes. We used *k*-means to cluster yeast genes into 100 different clusters based on their GIT-scores in the presence of different compounds. For each cluster, we used Fisher’s exact test to test whether it was enriched with the annotated genes of at least one Gene Ontology term. We obtained Gene Ontology annotations from BioGRID V3.4 [41]. We compared the clustering performance of using the GIT-score with using the FD-score from the HIP assay and using the FD-score from the HOP assay on three Gene Ontology categories in Fig 7 and S6 Fig. We can see that the GIT-score discovers more established complexes than the FD-score. For example, 93 of the 100 clusters identified by the GIT-score are significantly enriched with at least one cellular component function by using a false discovery rate of 0.005. In contrast, the FD-score from the HOP(HIP) assay only identifies 75(65) clusters that are significantly enriched with at least one cellular component function. In addition, the GIT-score also exclusively identifies many important cellular component complexes such as pore complex, chromosome, centromeric region, and microtubule.

## Discussion

Here we have reported the discovery that, through the use of prior knowledge captured in the genetic interaction network, compound-target interactions can be identified more accurately on chemical genomic screens. Our method identifies many compound-target interactions that comprise existing curated database as well as novel compound-target interactions that are supported by literature evidence. Due to its ability in modeling the genetic interaction among genes, we can better understand the mechanism of action of compounds, which may provide new insight into drug discovery and drug repositioning. Historically, genetic interaction profiles have been integrated with chemical genomic screens to identify compound-target interactions via the Pearson correlation coefficient [23] due to the inherent similarity between genetic perturbation and chemical perturbation. Our study is different from these previous works in that different local network topology features are taken into consideration in HIP and HOP assays. To the best of our knowledge, this is the first time that HIP and HOP assays are used differently to decipher compound-target interactions.

One might consider at least three potential reasons for the good performance of GIT. First, existing high-throughput chemical genomic screens might be noisy. GIT is more robust to the noise by using genetic interaction neighbors’ FD-scores to assist the inference of drug targets. Second, HOP assay and HIP assay are fundamentally different biological assays, thus prioritizing different sets of genes. We use direct neighbors’ FD-scores to identify compound-target interactions in the HIP assay, whereas we consider two-hop neighbors’ FD-scores to capture the drug target pathway buffer effect in the HOP assay. Moreover, combining predictions from the HIP assay and the HOP assay further makes GIT more robust. Finally, the genetic interaction network reflects the consequence of perturbing gene function and uncovers broader relationships between diverse functional modules, thus provides functional information that is largely invisible to physical interactions.

One interesting observation is that GIT achieves a substantial improvement in comparison to the *ρ*-score. Both the *ρ*-score and the GIT-score use genetic interactions to assist target identification. However, the *ρ*-score prioritizes genes according to the Pearson correlation between one gene’s chemical genomic profile and its genetic interaction profile. Consequently, it is sensitive to the noise in SGA and chemical genomic screens. In contrast, GIT scores a gene according to the dot product between its neighbors’ FD scores and their genetic interaction edge weights, making it more robust to the noise. More importantly, in HOP assays, unlike the *ρ*-score which only considers one gene’s one-hop genetic interaction neighbors, we also consider its two-hop genetic interaction neighbors. Our observation that the GIT^HOP^-score (two-hop) has much better performance than the GIT^HIP^-score (one-hop) and the *ρ*-score (one-hop) in HOP assays demonstrates the promising of considering two-hop genetic interaction neighbors in the HOP assay.

Finally, we see many opportunities to improve upon the basic concept of GIT in future work. First, although the current GIT framework is developed in an unsupervised fashion, the GIT-score can be used as the feature and plugged into off-the-shelf machine learning classifier for compound target identification on chemical genomic screens. Second, although this study focused on yeast chemical genomic assays, the GIT method is broadly applicable to any drug perturbation screens on other species [48]. Finally, current genetic interaction network is still noisy, whereas GIT can be potentially used to predict the genetic interaction given the compound-target interaction. For example, one gene with a low FD-score might have a negative genetic interaction with the drug target. In comparison to model organisms such as yeast and worm, high-throughput measuring genetic interactions in human is inherently difficult due to the lower efficiency of genetic engineering and the absence of resources like the yeast knockout collection. With available large-scale drug perturbation screens [48] in human, GIT offers the intriguing opportunity to explore genetic interactions in human.

## Supporting information

S1 Fig Comparison of GIT with other scoring methods in terms of nAUC_d_ in HIP assays on three chemical genomic screens. The y-axis shows the number of compounds, at least one target of which can be identified in the top *k* genes, where *k* is shown on the x-axis.
(EPS)

S2 Fig **A,C,E are the comparison of GIT with other scoring methods in terms of nAUC_t_ in HIP assays on three chemical genomic screens.** B,D,F are the comparison of GIT with other scoring methods in terms of nAUC_d_ in HIP assays on three chemical genomic screens. The GIT^HOP^-score is calculated by applying Eq 5 to HIP assay. The GIT^HIP^-score(PI) is calculated based on the physical interaction network instead of the genetic interaction network.
(EPS)

S3 Fig Comparison of GIT with other scoring methods in terms of nAUC_d_ in HOP assays on three chemical genomic screens. The y-axis shows the number of compounds, at least one target of which can be identified in the top *k* genes, where *k* is shown on the x-axis.
(EPS)

S4 Fig **A,C,E are the comparison of GIT with other scoring methods in terms of nAUC_t_ in HOP assays on three chemical genomic screens.** B,D,F are the comparison of GIT with other scoring methods in terms of nAUC_d_ in HOP assays on three chemical genomic screens. The GIT^HIP^-score is calculated by applying Eq 3 to HOP assay. The GIT^HOP^-score(PI) is calculated based on the physical interaction network instead of the genetic interaction network.
(EPS)

S5 Fig Venn diagrams show the overlap between the identified interactions by using the GIT^HIP^-score in the HIP assay and the identified interactions by using the GIT^HOP^-score in the HOP assay on (A) Hoepfner et al. 2014 and (B) Hillenmeyer et al. 2008, respectively. (EPS)

S6 Fig GIT identifies co-functional gene complexes in molecular function category. The y-axis shows the number of co-functional gene complexes that can be identified by the GIT-score, the FD-score from the HIP assay, and the FD-score from the HOP assay by using false discovery rate *r*, where *r* is shown in the x-axis.
(EPS)

S7 Fig The robustness of the result on different *q* values on three chemical genomics screens. The y-axis is the nAUC_t_. The x-axis is different *q* values. We show the nAUC_t_ of GIT^HIP^-score by using different *q* values.
(EPS)

S8 Fig The results of using *k*-hop neighbors where *k* is ranged from 1 to 10. The y-axis is the nAUC_t_. The x-axis is different *k* values. We show the nAUC_t_ of GIT^HOP^-score by using different *k* values. We found that *k* = 2 has the best overall performance on HOP assays. When *k* is larger, the performance becomes worse due to the increasing noise from long distance neighbors in the genetic interaction network.
(EPS)
